# Beyond the Usual Suspects: Unmasking Low-T2 Asthma in Children

**DOI:** 10.3390/jcm15020907

**Published:** 2026-01-22

**Authors:** Iva Mrkić Kobal, Marta Navratil, Helena Munivrana Škvorc, Andrija Miculinić, Davor Plavec

**Affiliations:** 1 Children’s Hospital Srebrnjak, 10000 Zagreb, Croatia; imrkic@bolnica-srebrnjak.hr (I.M.K.);; 2 Medical Faculty, University of Osijek, 31000 Osijek, Croatia; 3 Children’s Hospital Zagreb, 10000 Zagreb, Croatia; 4 University North, 42000 Varaždin, Croatia; 5 Prima Nova, 10000 Zagreb, Croatia

**Keywords:** T2-low asthma, biomarkers, children, endotype, neutrophils, therapy

## Abstract

Background: T2 low asthma in children is an emerging yet underexplored endotype that challenges traditional views of type 2 inflammation. Recent data suggest that it is more prevalent than previously thought and is defined by low type 2 biomarkers, non-allergic clinical profiles, and strong associations with modifiable comorbidities such as obesity, passive smoke exposure, and recurrent respiratory infections. This phenotype often shows a poor response to standard inhaled corticosteroid therapy and T2-targeted biologics, underscoring the urgent need for improved diagnostic and therapeutic approaches. Methods: This narrative review conducted a literature search from PubMed and WoS databases (2020–2025), focusing on T2-low asthma defined by low blood eosinophils (<150–300/µL), FeNO (<20–25 ppb), and absent atopy in children under 18. Results: This review highlights the heterogeneity of T2-low asthma, including subtypes from neutrophilic/Th 17-high to paucigranulocytic airway remodeling and metabolic driven forms, as well as diagnostic challenges from biomarker supresssion by high-dose therapies. Pragmatic phenotyping algorithms using routine tests enable identification, directing comorbidity management over ineffective biologics. Conclusions: Systematic T2-low phenotyping in pediatric practice, alongside prospective studies and non-T2 therapy trials, promises precision medicine to enhance outcomes for these children, moving beyond eosinophil-centric care.

## 1. Introduction

Asthma has long been recognized as a chronic airway inflammatory disease, with a prominent role attributed to eosinophils and the T2 immune response [1]. This paradigm, focused on eosinophil subtypes, has led to therapeutic breakthroughs and the development of biological agents (anti-immunoglobulin (Ig)E, anti-interleukin (IL)5, and anti-IL4 receptor (R)) [2]. These innovations have significantly improved outcomes for patients with severe T2-high asthma. Clinical practice and large cohort studies have shown that eosinophil-driven asthma responds best to corticosteroid and biologic therapy [3,4].

However, a growing body of research and clinical experience highlights the heterogeneity of asthma, which extends well beyond the traditional focus on eosinophils [5]. Asthma is not a single disease, but rather an umbrella term encompassing a group of clinical syndromes with multiple pathogenic pathways (endotypes), phenotypic variations, and distinct immune characteristics [6]. The new “omics era”—characterized by the systematic use of transcriptomics, proteomics, and other advanced technologies—has revealed a wide range of inflammatory profiles. Among these, particularly in children, rarely identified or previously “masked” forms such as T2-low asthma are beginning to emerge [7].

T2-low asthma (non-eosinophilic, often neutrophilic or paucigranulocytic asthma) is receiving increasing attention for several reasons. In large population analyses and among schoolchildren, T2-low asthma accounts for a significant proportion of asthma patients—according to some authors, up to 50% [8,9]. These patients often lack a response to standard therapy and often have comorbidities such as obesity or metabolic syndrome, or are exposed to environmental influences, especially in children; existing diagnostic strategies and treat-to-target approaches largely fail to identify these patients due to a lack of practical and reliable biomarkers, making timely and personalized treatment more difficult [7,10,11].

Recent research indicates that the presence of mixed or overlapping inflammatory profiles (T2 and non T2 pathways), along with complex mechanisms at the levels of the epithelium, microbiota, systemic inflammation, and other low-grade processes, calls for a redefinition of diagnostic, prognostic, and therapeutic paradigms in asthma [12]. Additionally, biomarker-guided approaches, important for T2-high asthma, still have not found a clear position in the recognition and treatment of T2-low and atypical forms of asthma. The complexity of these non-classic phenotypes emphasizes the need for the development of new diagnostic tools regarding the whole spectrum of the immunological mechanism, epithelial barrier, microbiome dysregulation, and metabolic abnormalities [10,13].

Therefore, the aim of this narrative review is to present the clinical and pathophysiological diversity of asthma with a focus on T2-low asthma and the “masks” of non-eosinophilic inflammation, diagnostic challenges in T2-low asthma, and the latest insights into the mechanism, clinical significance, and treatment possibilities of “beyond eosinophils”, particularly in children and adolescents.

## 2. Materials and Methods

To achieve our aim of investigating the characteristics, biomarkers, pathophysiology, and therapeutic approaches for type 2-low (T2-low) asthma in children, we conducted a narrative literature search in PubMed and Web of Science databases. Papers were identified using the following keywords in various combinations: “children” OR “pediatric OR pediatric” AND “asthma” AND “T2 low “ OR “T2-low” OR “type 2 low” OR “non-type 2 asthma” AND “therapy” OR “treatment”, AND “biomarkers” AND “pathophysiology” AND “endotype”.

### 2.1. Inclusion Criteria

- Peer-reviewed articles in English (2020–2025);
- Pediatric population (age < 18 years);
- Explicit T2-low asthma definition (e.g., blood eosinophils < 300 cells/μL and/or FeNO < 25 ppb);
- Primary data on biomarkers, pathophysiology, endotypes, or therapeutic interventions.

### 2.2. Exclusion Criteria

Titles and abstracts were screened (321 total: 227 PubMed and 94 WoS), excluding irrelevant studies. A total of 55 full texts were assessed, excluding case reports, non-pediatric studies, ambiguous T2-low definitions, and non-English publications. Disagreements were resolved by author consensus.

Overall, a total of 22 articles were included in this narrative review. We included one epidemiological study pre-2020 from references due to scarce pediatric T2-low asthma data, for clinical relevance.

## 3. Results

### 3.1. Shifting Paradigms: From T2 High to T2 Low

Asthma phenotyping began in the late 1940s, when Rackemann [14] identified two distinct types of the disease: “extrinsic” asthma, primarily linked to atopy, and “intrinsic” asthma, which occurs without atopic features. These early efforts to phenotype asthma relied solely on a patient’s clinical and functional features, with no attention given to the underlying molecular profiles [15]. At the end of the 1990s, Wezel et al. defined two different asthma phenotypes: eosinophilic and neutrophilic [16]. In 2008, Anderson introduced the concept of asthma endotypes, to understand different forms of the disease. The concept of an endotype emerged as an idea of a single pathway underlying all clinical features in a single phenotype. The goal of endotyping, according to Anderson, was to link biological mechanisms to clinical manifestations to develop more precise diagnostics and targeted therapeutic approaches. Rather than viewing asthma as a single condition, he pointed out that there are different pathophysiological mechanisms (e.g., eosinophil inflammation, neutrophils, epithelial dysfunction, airway hyperresponsiveness) that lead to similar clinical symptoms [17]. One well-defined asthma endotype, known as classical T2-high asthma, has been the focus of extensive research, particularly in pediatric and adolescent populations. This endotype is distinguished by elevated activity of type 2 cytokines (IL-4, IL-5, and IL-13), which drive airway eosinophilia and allergic inflammation. Clinically, T2-high asthma is often identified through increased serum total immunoglobulin E (IgE), elevated blood eosinophil counts, higher fractional exhaled nitric oxide (FeNO) levels, and good response on corticosteroid treatment [18]. T2-low asthma is a biologically distinct endotype that differs from the classical T2-high inflammatory pattern. It is characterized by reduced activation of type 2 immune pathways and consequently low levels of biomarkers such as interleukins IL-4, IL-5, and IL-13, total IgE, blood and airway eosinophils, and fractional exhaled nitric oxide (FeNO) [19]. However, there is a total lack of consensus on diagnostic criteria for T2-low asthma, particulary eosinophil counts, as T2-low is defined negatively by the absence of T2-high features using inconsistent thresholds (e.g., blood eosinophils < 150–300 cells/µL or <470 cells/µL/90th percentile, varying by age and study). Blood eosinophils poorly correlate with airway eosinophilia in children, especially in severe cases, further complicating reliable classification. A significant selection bias arises from cohorts often comprising patients on inhaled corticosteroids (ICS), which suppress eosinophil counts and inflate “false positive” T2-low prevalence (e.g., up to 36.5% ICS use in preschool wheezers), masking underlying T2-high disease [20,21,22]. Based on inflammatory activity and cellular response, we differ several subtypes of T2-low asthma: neutrophilic, paucigranulocyte, mixed granulocyte asthma, and asthma related to obesity [23]. An endotype may be conceptualized as a “hidden subtype” of asthma, one whose biological identity often remains obscured amidst clinical complexity. The additional challenge remains the fact that these biological subtypes are not fixed; they can shift or overlap over time in response to various internal and external influences. For instance, a child’s underlying endotype may remain “masked” until a change in therapy, a shift in environmental exposure, or the development of a new immune response unmasks a different inflammatory mechanism [24].

### 3.2. Spotlight on T2 Low Asthma in Children

The reported prevalence of T2-low asthma differs widely across studies due to variations in its definition, the populations examined, and the diagnostic criteria applied, making direct comparison of the findings challenging [25]. A study conducted in the United States analyzed data from the National Health and Nutrition Examination Survey (NHANES) from 2007 to 2012, including 4284 children aged 6 to 17 years. According to the results, 45.7% of children with asthma had T2-low asthma, defined as a blood eosinophil count of less than 300 cells/µL and a FeNO level of less than 25 ppb. These data suggest that T2-low asthma is a significant endotype among children with asthma in the United States [20]. The ALLIANCE cohort study represents one of the most comprehensive studies to analyze the distribution of T2-inflammatory asthma phenotypes across different age groups, including children, adolescents, and adults. It is a multicenter study conducted by the German Lung Research Center (DZL), with a total of 1125 participants, of whom 776 had asthma and 349 were healthy controls without a diagnosis of asthma. The following phenotypes were defined: T2-high (elevated eosinophils and allergic sensitization), eosinophilic without atopy, atopic without eosinophilia, and T2-low (no eosinophilia and no sensitization). The threshold for eosinophilia in children was set at ≥470 cells/µL, while for adults it was ≥360 cells/µL. The results of the ALLIANCE study indicate that T2-inflammatory phenotypes dominate in childhood asthma, while T2-low asthma represents an important clinical entity that requires a specific therapeutic approach. The prevalence of T2-low asthma in the study ranged from 11.9% to 64.8%, depending on the age group of the children. The prevalence rates were higher in infants and preschool children. These findings further emphasize the need for a phenotypic and endotypic approach to the diagnosis and treatment of children with asthma [21]. A retrospective study conducted in China analyzed data of children and adolescents with asthma who were hospitalized for asthma exacerbations in the period from January 2016 to December 2021. The children were classified into four asthma phenotypes: Only-atopy, Only-EOS, T2-high, and T2-low groups based on their blood EOS count and sIgE results before or after 3 days of admission. Different eosinophil thresholds have been proposed to stratify asthma severity and T2 inflammation, with 150 cells/μL, 300 cells/μL, and 470 cells/μL. The prevalence rates of T2-low asthma were: 19.4%, 25.6%, and 28.2%, according to EOS count [22]. Overall, current evidence suggests that T2-low asthma represents approximately 30–50% of asthma cases, underscoring its clinical relevance and the need for tailored therapeutic approaches (Table A1).

### 3.3. Beneath the Surface: Mechanism and Biomarkers

#### 3.3.1. Risk Factors and Pathophysiological Modulators of T2-Low Asthma in Children and Adolescents

(1) Environmental Exposures and Epithelial Injury as Early Drivers of T2-Low Asthma

Environmental exposures appear to play a predominant role in the early development and priming of T2-low asthma in children, establishing a foundation for non-eosinophilic, neutrophil-dominant airway inflammation [26]. Environmental risk factors for T2-low asthma include older age, female gender, obesity, exposure to tobacco smoke, poorer lung function, and frequent respiratory infections in early life [20,27]. Chronic exposure to air pollutants, including nitrogen dioxide (NO_2_), ozone (O_3_), and fine particulate matter (PM_2.5_), has been shown to directly injure airway epithelial cells and compromise barrier integrity. This dysfunction not only facilitates the penetration of environmental agents and microbial products but also triggers the release of epithelial-derived “alarmins” and pro-inflammatory cytokines such as IL-1β, IL-6, and TNF-α [28]. These mediators subsequently activate Th1 and Th17 immune pathways and promote neutrophil recruitment and activation, hallmarks of T2-low asthma endotypes. In parallel, recurrent viral infections during early life—particularly with rhinovirus, respiratory syncytial virus (RSV), and influenza—can skew immune maturation toward type 1 and type 3 responses, suppressing T2 polarization and driving non-eosinophilic inflammation [29,30]. This deviation may be further amplified by alterations in the microbiome due to antibiotic exposure, urban living, or reduced microbial diversity, all of which have been linked to a loss of immune tolerance and the emergence of T2-independent airway disease [31,32,33].

(2) Hormonal and Pubertal Modulation of the T2-Low Asthma Phenotype

While environmental exposures initiate T2-low inflammatory pathways early in life, pubertal and hormonal changes act as key modulators that influence the persistence, phenotype, and treatment response of asthma during adolescence. Puberty represents a transitional period characterized by profound endocrine, metabolic, and structural changes, all of which interact with the immune system and airway physiology. In a NHANTES study, children with T2-low asthma were found to be 1.7 to 2.1 times more likely to be female and to have a body mass index (BMI) z-score at or above the 85th percentile, compared with children with T2-high asthma [34]. The sex shift in asthma prevalence—from a male predominance in childhood to a female predominance after puberty—strongly suggests that sex hormones exert active immunomodulatory effects [35,36], alter barrier/epithelial function, and shift immune balance away from classic Th2 pathways—potentially favoring alternative (T1/T3) routes of inflammation [35,37]. Estrogens and progesterone jointly upregulate IL-17A expression in Th17 cells via a Let-7f/IL-23R-dependent pathway [38]. Elevated IL-17A levels correlate with neutrophilic inflammation, a hallmark of corticosteroid-resistant asthma, indicating that estrogen’s role extends beyond classical type 2 inflammation [39]. Conversely, studies in allergic mouse models demonstrated that testosterone treatment reduces neutrophilic inflammation [40]. Beyond immunological effects, estrogen also modulates airway epithelial function [41] and glucocorticoid receptor signaling [42]. Notably, sex differences in airway dimensions appear to result primarily from hormonal changes during puberty and occur independently of height, supporting a mechanistic link between pubertal development and asthma susceptibility [43]. Collectively, these findings suggest that pubertal hormonal changes function as amplifiers rather than initiators of T2-low inflammation, reinforcing the neutrophilic, non-eosinophilic phenotype established by earlier environmental exposures. The combined impact of environmental priming and hormonal modulation likely underlies the persistence, severity, and steroid insensitivity characteristic of T2-low asthma in adolescent females.

#### 3.3.2. Potential Mechanisms of T2-Low Asthma

Several distinct mechanisms have been proposed to underlie T2-low asthma, reflecting its complex pathophysiology and poor responsiveness to conventional corticosteroid therapy [44].

(1) Non-T2 Inflammation in the Lung: Neutrophilic Asthma

One of the primary mechanisms involves T2-low inflammation within the airways, typically marked by neutrophilic infiltration. This inflammatory pattern is often associated with a type 1 (T1) immune response mediated by interferons (IFNs), or type 3 (T3) immune pathways driven by interleukin-17 (IL-17). As previously described, these immune responses can be triggered by infections, environmental pollutants, or microbiome alterations, contributing to persistent airway inflammation and remodeling even in the absence of eosinophilic activity [45].

(2) Role of Neutrophils and Neutrophil Extracellular Traps (NETs) in T2-Low Asthma

Neutrophils, the most abundant cells of the innate immune system, play a central role in host defense through mechanisms such as phagocytosis, degranulation, and the formation of neutrophil extracellular traps (NETs). NETs are web-like structures composed of DNA and granule proteins that trap and kill pathogens, including bacteria and viruses, thereby preventing their dissemination [46]. Airway neutrophilia has been frequently observed in patients with more severe and chronic forms of asthma, and is a hallmark feature of the T2-low phenotype. Although neutrophilic inflammation can occur independently of eosinophilia, the presence of both (mixed granulocytic inflammation) is often associated with particularly severe disease. Neutrophilia is also commonly seen during asthma exacerbations and is believed to contribute to epithelial injury through several mechanisms: the release of proteolytic enzymes, induction of oxidative stress, stimulation of goblet cell degranulation, and NET formation [47]. NETs can disrupt the integrity of the bronchial epithelium by damaging tight junctions, leading to the leakage of intracellular components. Through direct interaction with bronchial epithelial cells (BECs), NETs stimulate the secretion of inflammatory mediators that enhance airway inflammation and contribute to respiratory symptoms [48,49,50]. Specific components of NETs, such as high-mobility group box 1 protein (HMGB1), can stimulate BECs to produce mediators implicated in asthma pathogenesis, including TSLP, TNF-α, MMP-9, and VEGF. Consequently, NETs may play a role in asthma development and exacerbations by compromising bronchial epithelial barrier integrity and inducing the release of upstream cytokines, or alarmins, such as TSLP and IL-33 [51]. Moreover, various bacterial components (e.g., formyl-methionyl-leucyl-phenylalanine, fMLP) [52] as well as viral pathogens such as rhinovirus and influenza virus [53] have been shown to induce the formation of NETs. Microbe-induced NETs activate inflammasome signaling and stimulate IL-1β release, which in turn further promotes NET formation, creating a self-amplifying loop of neutrophilic inflammation central to the pathogenesis of T2-low asthma. However, accumulating evidence suggests that virus-induced NET formation may drive airway inflammation that cannot be strictly classified as either T2-high or T2-low. In this context, recent studies have revealed a paradoxical effect in which rhinoviral infections trigger NET release that can unexpectedly enhance T2 immune responses [53]. This observation supports the concept that inflammatory endotyping in asthma is influenced by multiple overlapping factors acting in concert, rather than by a rigid dichotomy, ultimately shaping a mixed or context-dependent inflammatory phenotype [54]. The NET/IL-17A axis plays an important pathogenic role in asthma exacerbation, linking airway inflammation to fibroblast dysfunction and fibrosis [55].

(3) Neutrophilic Asthma

Neutrophilic airway inflammation is commonly associated with environmental exposures, such as air pollutants, as well as with infections and bacterial colonization, both of which can exacerbate inflammation and contribute to increased disease severity [56]. Chronic bacterial colonization and recurrent infections constitute an additional important factor driving neutrophilic airway inflammation in T2-low asthma. Patients with T2-low asthma frequently exhibit reduced airway bacterial diversity and an increased presence of respiratory pathogens, particularly *Moraxella* and *Haemophilus* species. In a bronchoscopy study of 126 children with severe asthma, 15.9% showed isolated neutrophilia in BAL, of whom 65% had detectable respiratory pathogens [57]. Lung microbiome dysbiosis, including persistent colonization by non-typeable *Haemophilus influenza* [58], has been associated with the neutrophilic phenotype, suggesting a potential therapeutic role for antibiotics such as azithromycin. Patients experiencing non-eosinophilic asthma exacerbations, characterized by low FeNO levels (≤20 ppb) and higher bacterial load, may derive greater benefit from antimicrobial therapy compared with those with eosinophilic exacerbations, who typically exhibit elevated FeNO levels (≥50 ppb) and a lower bacterial burden [59].

(4) Non-T2 Immune Pathways in T2-Low Asthma: Type 3 and Type 1 Immunity

Epithelial injury contributes to non-eosinophilic, T2-low asthma by compromising the airway barrier and facilitating the entry of environmental agents. This epithelial dysfunction triggers the release of alarmins and pro-inflammatory cytokines (IL-1β, IL-6, and TNF-α), which activate TH1 and TH17 cells, as well as ILC1 and ILC3. The resulting type 1 (IFN-mediated) and type 3 (IL-17–mediated) immune responses drive neutrophilic inflammation, steroid resistance, and more severe asthma phenotypes [28] (Figure S1).

Type 3 immunity is driven by the IL-17 cytokine family, particularly IL-17A, which is secreted by a variety of immune cells, including Th17 cells, γδ T cells, CD8^+^ T cells, natural killer T cells, and subsets of innate lymphoid cells (ILCs), such as ILC3s. IL-17A has several pathogenic roles in the asthmatic airway:

- Promotion of neutrophilic inflammation via the induction of granulocyte colony-stimulating factor (G-CSF) and neutrophil-attracting chemokines.
- Contribution to corticosteroid resistance [60].
- Enhancement of airway hyperresponsiveness [61,62].

Animal models have shown that allergic sensitization through the airway or skin can induce bronchial Th17 responses and airway hyperreactivity [63]. In humans, elevated levels of IL-17A or IL-17–producing cells have been observed in blood, sputum, and bronchial biopsies of patients with severe asthma [64]. Despite promising observational data, clinical trials targeting IL-17 have not yet yielded clear benefits. A phase 2 trial of brodalumab, an anti–IL-17 receptor antibody, did not improve asthma control across unselected patients [65]. These results highlight the need for more precise patient stratification, possibly using IL-17–related biomarkers. Interestingly, there is emerging evidence that suppression of T2 pathways may unmask or even promote IL-17-driven inflammation, suggesting that dual targeting of T2 and IL-17 pathways could be a rational approach for certain patients [66].

Type 1 immunity, primarily directed against intracellular pathogens, involves IFN-γ–producing cells such as Th1 CD4^+^ T cells, CD8^+^ T cells, NK cells, and ILC1s [67]. Additionally, airway epithelial cells contribute to type 1 responses through the secretion of type I (IFN-α, IFN-β) and type III (IFN-λ) interferons, especially in response to viral infections and some bacteria [68,69]. The role of IFNs in asthma is complex and appears to be context-dependent:

- Deficient IFN signaling, particularly in the airway epithelium, has been reported in some patients with asthma [70], potentially increasing susceptibility to viral infections and asthma exacerbations [71].
- However, increased IFN-γ expression has also been observed in patients with severe asthma, especially in those with steroid-resistant disease. Elevated IFN-γ levels in bronchoalveolar lavage fluid correlate with markers of airway inflammation and mast cell activation, such as increased CXCL10 expression [72] and IFN regulatory factor 5 (IRF5) [73].

To date, randomized trials of inhaled interferon therapy have failed to demonstrate significant improvements in asthma symptoms or reductions in virus-induced exacerbations [74]. In children with severe, treatment-refractory asthma, airway inflammation is dominated by memory CCR5^+^ Th1 and Th17-type responses, with CD8^+^ T cells primarily producing IFN-γ. Despite low Th2 cell numbers, Th2 cytokines were detectable and correlated with total IgE, particularly in multi-sensitized children. Innate type 2 cells (ILC2s) and basophils were scarce in BAL fluid, while plasmacytoid and IgE^+^FcεRI^+^ myeloid dendritic cells were consistently present. Cytokine profiles, including IL-5, IL-33, and IL-28A/IFN-λ2, were associated with allergen sensitization patterns, age, and eosinophil counts, highlighting a complex interplay of Th1, Th17, and selective Th2 responses in the airway immune environment of pediatric severe asthma [75].

(5) Systemic Inflammation

Several factors, including obesity, metabolic syndrome, aging, and inflammaging, contribute to systemic inflammation in adult patients with T2-low asthma [10]. These systemic factors can exacerbate airway hyperresponsiveness and influence the immune milieu of the lungs, even in the absence of overt local inflammation. In children, systemic inflammation appears to be less pronounced and differently regulated compared with adults, and thus likely contributes differently to the development of T2-low asthma. In adults, obesity is strongly associated with chronic low-grade systemic inflammation, which contributes to T2-low asthma via M1 macrophage-mediated secretion of IL-1β, IL-6, and TNF-α in adipose tissue, promoting airway neutrophilia and more severe disease. Elevated CRP, IL-6, and leptin in adults are also linked to alterations in both airway and systemic immune profiles, correlating with worse asthma outcomes [76]. In children, while obese asthmatic patients may exhibit low-grade systemic inflammation (e.g., elevated leptin, IL-6, and TNF-α), the magnitude and impact of this inflammatory burden are generally lower than in adults. In adolescents, obesity and asthma are independently and synergistically associated with elevated hs-CRP, indicating some contribution of systemic inflammation, but it remains less robust than in adults [77]. In adolescents, obesity and asthma were independently and synergistically associated with elevated high sensitivity (hs)-CRP, a marker of systemic inflammation [78]. In children, developmental mechanisms refer to non-inflammatory processes governing early lung and airway maturation that may predispose asthma independent of classic T2 inflammation. During early life, structural airway changes—such as increased reticular basement membrane thickness, airway smooth muscle hyperplasia, and vascular remodeling—are observable even in the absence of marked eosinophilic inflammation [79]. These alterations suggest that airway remodeling can precede overt inflammation and thus may represent a distinct mechanistic pathway in childhood asthma [80]. Moreover, immune ontogeny in early childhood appears to differ from that in adults—neonatal and preschool-aged subjects show a preferential reliance on IL-13^+^ CD4^+^ T cells for the initiation of airway hyperresponsiveness, rather than innate ILC2-driven responses typical of older individuals [81]. In a cohort of 105 children aged 1–5 years with recurrent severe wheeze [82], four distinct clusters were identified: atopic (23.1%), non-atopic with low infection and high ICS use (36.5%), non-atopic with high infection and highest BAL neutrophils (21.2%), and non-atopic with low infection and low ICS use (19.2%). Notably, most children (76.9%) fell into the non-atopic clusters, highlighting the potential for early-life, T2-low or non-eosinophilic phenotypes to contribute to persistent asthma later in childhood. These observations underscore that mechanisms driving severe asthma and its risk factors differ between adults and children, with early-life T2-low phenotypes potentially representing a fundamentally different pathophysiology compared with adults [79,80,81].

(6) Non-Inflammatory (Paucigranulocytic) Mechanisms

A subset of T2-low asthma cases exhibits a paucigranulocytic phenotype, defined by the absence of both eosinophilic and neutrophilic inflammation. In such cases, non-inflammatory mechanisms—including airway smooth muscle dysfunction, neural dysregulation, and altered epithelial barrier function—may contribute to clinical symptoms [83]. Airway efferent nerves, controlled by parasympathetic cholinergic neurons, mediate airway smooth muscle (ASM) contraction and contribute to airway hyperresponsiveness (AHR). In mice, nerve growth factor (NGF) administration induces AHR comparable to allergen exposure without airway inflammation [83,84]. Human studies show increased cholinergic nerve density and TrkB expression in asthmatic airways, independent of eosinophil levels [85]. Genetic factors can also dissociate AHR from inflammation. Polymorphisms in 17q21 leading to ORMDL3 overexpression reduce sphingolipid synthesis, increase AHR, and promote airway remodeling without inflammation [86]. T2-low asthmatic children show lower serum sphingolipids than T2-high or non-asthmatic children [87]. GPCR signaling regulates ASM contraction via Gαq-mediated calcium influx, while RGS proteins terminate signaling. Reduced RGS2/5 expression in asthma or knockout mice leads to AHR independently of inflammation [88]. Ongoing studies aim to clarify the role of RGS dysregulation in paucigranulocytic asthma. In adult patients, paucigranulocytic asthma (PGA) is typically associated with well-controlled disease and preserved lung function under optimal therapy, including low-dose inhaled corticosteroids (ICS). In this population, PGA may predominantly represent the therapeutic resolution of previously eosinophilic asthma rather than a distinct inflammatory endotype. It is generally characterized by lower levels of inflammatory biomarkers and a reduced requirement for high-dose ICS [89]. In contrast, data from a large bronchoscopy study of 126 children with severe asthma showed that 52% exhibited a paucigranulocytic pattern, characterized by less post-bronchodilator airflow limitation, lower blood eosinophilia, and less frequent pathogen detection [57]. This high prevalence suggests that, in children, paucigranulocytic asthma may reflect a distinct pathophysiological mechanism, potentially differing from the largely treatment-responsive phenotype observed in adults.

#### 3.3.3. Biomakers

T2-low asthma is a heterogeneous subtype of asthma defined primarily by the absence of T2-mediated inflammation and its associated biomarkers, including eosinophils and T2 cytokines such as IL-4, IL-5, and IL-13, rather than by the presence of any specific low T2 marker. In clinical trials, it is often characterized by a blood eosinophil count (BEC) of less than 150 cells/μL and fractional exhaled nitric oxide (FeNO) levels below 20–25 parts per billion (ppb). However, these biomarkers can be highly variable and are susceptible to suppression by corticosteroid therapy, which complicates accurate classification. As a result, a reliable diagnosis may require a multidimensional approach that includes multiple biomarkers and repeated longitudinal assessments. Despite these efforts, the identification of T2-low asthma using clinically accessible and validated biomarkers remains a significant unmet need. Immunologically, T2-low asthma is associated with alternative inflammatory pathways involving T helper 1 (Th1) and T helper 17 (Th17) cells, neutrophilic airway inflammation, and increased expression of pro-inflammatory cytokines, including interleukin (IL)-1β, IL-6, IL-8, and IL-17A/F, and interferon-gamma (IFN-γ) [44,76]. Recent studies have shown the heterogeneity of T2-low asthma, identifying nine molecular clusters distinguished by combinations of enrichment scores (ES) across different pathways, including T2-high and T2-low pathways, which were associated with variations in clinical and inflammatory characteristics. T2-low asthma was found to be heterogeneous, comprising eight distinct clusters, whereas T2-high asthma appeared relatively homogeneous. The inclusion of two proteomic platforms in the analysis allowed for greater granularity within the T2-low clusters. The presence of multiple T2-low phenotypes poses challenges for the identification of specific biomarkers and the development of targeted therapies, in contrast to the single T2-high cluster. By integrating sputum transcriptomic and serum proteomic analyses, these studies identified distinct molecular subgroups within the T2-low asthma population. The cohort consisted of adult patients with clinically characterized T2-low asthma, defined by low Type 2 inflammatory biomarkers and an absence of eosinophilic airway inflammation. This integrative multi-omics approach demonstrated that T2-low asthma is not a homogeneous phenotype but comprises several biologically distinct endotypes, each characterized by unique gene-expression and protein-biomarker signatures. These findings underscore the value of combining sputum and serum molecular profiling to improve disease stratification and inform the development of more targeted therapeutic strategies for non-Type 2 asthma [90].

Potential T2-low asthma biomarkers in children are presented in Table A2.

### 3.4. Clinical Manifestations: When Typical/Usual Signs Go Missing

While T2-high asthma is predominant among patients with severe asthma, T2-low phenotypes appear to be more commonly observed in individuals with mild to moderate disease. Notably, in patients with severe T2-low asthma, disease exacerbations may be associated with a temporary shift toward a T2-high inflammatory profile, indicating a dynamic and potentially fluctuating immunological response [76]. It is also important to note that oral corticosteroids significantly reduce blood eosinophil counts (BEC) and moderately suppress FeNO levels, which may contribute to the overdiagnosis of T2-low asthma following corticosteroid rescue treatment during exacerbations [76,91]. It is well established that corticosteroid treatment during asthma exacerbations can induce a shift from a T2-high to a T2-low inflammatory profile. This corticosteroid-driven modulation of inflammation partly explains the relatively low prevalence of T2-low asthma observed in patients with severe asthma, as many present with T2-high features during exacerbations but may appear T2-low under corticosteroid influence. Notably, a study showed that only 5% of patients with severe asthma maintained a T2-low profile after tapering oral or inhaled corticosteroids [92]. In contrast, among patients with mild to moderate asthma who were corticosteroid-naive, approximately 40.4% exhibited a T2-low phenotype defined by the absence of elevated sputum eosinophils [93]. These findings highlight that T2-low asthma is more predominant in mild to moderate asthma compared to severe asthma, where corticosteroid use and disease severity mask the true immunological phenotype. This underscores the dynamic and fluctuating nature of inflammatory pathways in asthma and the need for careful phenotyping, particularly in the context of corticosteroid exposure. Even though distinct lung-function patterns by endotype are lacking, conventional tests remain essential for monitoring T2-low asthma. Spirometry assesses airflow obstruction through the FEV1/FVC ratio, while FEF25–75% is particularly sensitive to small-airway disease. Impulse oscillometry is especially useful in young children, measuring airway resistance (R5–R20) and reactance area (AX) to detect early abnormalities. Peak expiratory flow diaries identify variability greater than 20%, helping to inform treatment escalation. Together, these functional measures complement biomarker assessment, since T2-low asthma shows a degree of impairment similar to T2-high disease but responds less well to inhaled corticosteroids [92]. Comorbidities frequently associated with T2-low asthma—such as obesity, depression, and anxiety—may, in many cases, arise because of inadequate disease control and prolonged exposure to ineffective therapies. Due to their reduced responsiveness to corticosteroids and short-acting β_2_-agonists (SABAs) [44,94], patients with T2-low asthma often receive stepwise treatment intensification according to current guidelines, which primarily rely on escalating corticosteroid doses. In T2-low asthma, this approach may lead to overtreatment with corticosteroids, offering limited therapeutic benefit while increasing the risk of treatment-related adverse effects. Notably, corticosteroid overuse has been linked to the development or worsening of obesity, depression, and anxiety [90], suggesting that these comorbidities may not only contribute to poor asthma outcomes but also reflect the consequences of suboptimal and non-targeted management strategies in this asthma endotype.

### 3.5. Therapy Reimagined: Management Strategies for Low-T2 Asthma

As it was already mentioned before, asthma is not a single disease, but a syndrome consisting of specific similar characteristics, namely airway inflammation, airway hyperresponsiveness, and episodes of bronchoconstriction, which all lead to the usual clinical manifestations of asthma (cough, wheezing, shortness of breath, etc.) [95]. Treatment options up until a few years ago were primarily focused on the most common type of asthma, with Th2 inflammation (eosinophilic asthma). In T2-low asthma, however, most medications targeting the Th2 inflammation pathway show little to no effect, and most patients with this type of asthma were deemed as having severe or difficult to treat asthma, although recent studies show that this might not be true [96]. Due to the fact that the diagnostic tools available today help clinicians to better define the underlying pathophysiologic mechanisms, the search for new, or rather targeted therapies, may be promising. In the next few paragraphs, we will try to shed light on current and potential treatment options and their mechanisms of action.

#### 3.5.1. Corticosteroids

Corticosteroids are among the most potent anti-inflammatory drugs. Because airway inflammation is the hallmark of asthma, corticoids should in theory always be useful. However, in T2-low asthma, corticosteroids, either inhaled or systemic, have shown poor asthma control. Additionally, prescribing higher doses (according to GINA step-up therapy and NICE guidelines) did not achieve any benefit, only the risk of more side-effects [97,98]. Oral corticosteroids have even been shown to be able to worsen T2-low asthma by means of promoting neutrophil accumulation and reducing their natural apoptosis, prolonging the inflammatory process of the lungs [99]. Therefore, corticosteroid therapy should not be considered as the main controlling medication, if mild to moderate doses do not achieve reduction in exacerbations.

#### 3.5.2. Bronchodilators

Bronchodilators, particularly short-acting beta-agonists (SABAs), remain a central component for rapid symptom relief and management of acute exacerbations across asthma inflammatory phenotypes, and are generally administered alongside controller medications. Long-acting beta-agonists (LABAs) are consistently prescribed only in fixed combination with inhaled corticosteroids (ICS) [100]. More recently, long-acting muscarinic antagonists (LAMAs) such as tiotropium bromide, glycopyrronium, and umeclidinium have emerged as additional bronchodilator options, and contemporary Global Initiative for Asthma (GINA) reports now include LAMAs as an add-on treatment for severe asthma in school-aged children and adolescents [97,101]. Clinical trial data indicate that tiotropium bromide, when added to existing controller therapy, improves lung function and is associated with reduced use of rescue medication in both school-aged children and adolescents with moderate-to-severe or severe asthma [102].

#### 3.5.3. Biologic Therapy

Most biologic agents are monoclonal antibodies (MABs) synthesized to selectively target and bind to a cell type or signal molecule, thus blocking the downwards path of their cascade of actions. This concept has revolutionized modern medicine, without which many immunologic, hematologic, endocrine, and other fields would be almost powerless [103,104]. As our focus is on asthma, we can mention a multitude of biologics, for instance omalizumab, dupilumab, mepolizumab, tezepelumab, etc. Omalizumab has, surprisingly, shown to reduce asthma exacerbations in both T2-high and T2-low asthma, although patients with eosinophillic asthma tend to benefit more (it lowers the biomarkers and also the effector cells) [105]. Mepolizumab (an IL-5 inhibitor) and dupilumab (an IL-4/IL-13Ra inhibitor), on the other hand, have not shown any benefit, as they target specific molecules on the T2 inflammation pathway [106,107]. Tezepelumab has already shown its efficacy in treating asthma, by other means difficult to treat, not only in T2-high, but also in the T2-low endotype. The explanation is simple—the target of this biologic drug is the thymic stromal lymphopoietin (TSLP), an inflammatory mediator above the T2-high and T2-low levels, thus leading to better control in both endotypes [108]. It is also approved for use in some countries for children with severe asthma above the age of 12, but there are ongoing trials examinig the efficacy and safety in preschool and school children between the ages of 5 to 12 years [109,110]. Clazakinumab, an anti-IL6 biologic agent, could be a promising candidate for some subgroups of patients with T2-low asthma, because some of them, especially older patients with metabolic syndrome (central obesity and diabetes), have been shown to have an increase in circulating IL-6 [110,111].

#### 3.5.4. Antibiotics

Of all the antibiotics, one specifically stands out—azithromycin. Some use it almost as a kind of “panacea” because it has shown promising effects on several diseases where inflammation plays a key pathophysiologic mechanism. Immunomodulation is the reason why this antibiotic drug shows more than only an antimicrobial function. It has shown beneficial effects in children with asthma, but without a direct effect on inflammatory markers [112,113]. Studies have shown that a long-term use of azithromycin three times a week has achieved symptom remission in adult patients with both T2-high and T2-low asthma [114]. However, the underlying mechanism is not well understood and certainly does not affect the reduction in T2-high asthma biomarkers. More studies are needed to define the optimal long-term low-dose regimen [115].

#### 3.5.5. Nonpharmacologic Treatment (Targeting Preventable Causes/Traits)

As T2-low asthma shares some characteristics with comorbidities, after extensive research has been performed, a connection between environmental factors and T2-low asthma has been established. The main causes of non-eosinophilic inflammation seem to be air pollution, smoking (active or passive), and obesity [9,23]. This is another proof that a healthy diet and microclimate have a huge impact on overall health, not only in patients with asthma, but also many other diseases. A cross-sectional study established a connection between T2-low asthma prevalence and obesity and cigarette smoke exposure [37,116]. Also, the same asthma endotype was more prevalent in countries with significant airway pollution. This is supposed to be the result of neutrophil inflammation activated by pathogen-associated molecular patterns [116]. In conclusion, therapy for T2-low asthma should be carefully adjusted according to several individual factors. Primarily following guidelines may be beneficial in mild to moderate asthma, but in severe cases or unresponsiveness to standard treatment, biologic agents like tezepelumab or long-term azithromycin may be considered. Finally, especially in high-risk children (like a positive family history of asthma, atopy, and recurrent wheezing episodes in early childhood), leading a healthy and unburdened lifestyle may be the best way to prevent later asthma development.

#### 3.5.6. Discussion—Toward Personalized Medicine: Future Directions

To achieve genuinely personalized care for pediatric asthma, future research must move beyond the simple T2-high/T2-low dichotomy and adopt multidimensional, longitudinal assessments at the level of each individual patient [117]. Evidence from pediatric studies indicates that inflammatory patterns are frequently heterogeneous and evolving, and are not fully reflected by existing biomarkers such as blood eosinophils and FeNO, particulary due to widespread corticosteroid suppression. The critical first steps include mandatory ICS withdrawal (>4 weeks) prior to biomarker assessment to unmask true endotypes, alongside standardized age-adjusted eosinophil cut-offs (<150–300 cells/µL) validated against airway inflammation. (Figure S2) This diagnostic challenge underscores the need for integrative approaches combining clinical, physiological, molecular, and environmental data. High-throughput analyses across genomics, transcriptomics, proteomics, metabolomics, and the microbiome offer an unprecedented opportunity to more precisely define pediatric asthma endotypes, including rare or “hidden” T2-low phenotypes. These technologies can uncover upstream regulatory circuits, airway epithelial signaling, and non-T2 inflammatory mechanisms that are not detected by conventional biomarkers such as blood eosinophils, FeNO, and IgE [118]. In doing so, they open the door to novel therapeutic targets for children who do not conform to classic T2-high profiles. Longitudinal omics studies, ideally using minimally invasive specimens such as nasal brushings, exhaled breath condensate, and stool, will be essential for monitoring endotype stability over time and in response to therapy, and for distinguishing short-term biomarker variability from stable disease trajectories [119]. To achieve personalized care in the future, we will have to integrate systemic inflammation, airway biology, and clinical status into composite risk scores relying on biomarker panels rather than single analyte cut offs. Creating and validating practical, age-specific biomarker algorithms that are feasible for routine clinical use—ideally based on blood testing and simple measures of lung function or symptoms—should be a major research priority, especially for low T-2 asthma endotypes. Biological therapy targeting epithelial alarmins represent a key step toward endotype-agnostic therapy, with tezepelumab already showing efficacy across both high and low T2 biomarker groups [120]. Future directions include refining which pediatric subgroups benefit most from such agents, investigating combinations or sequencing with existing T2 biologics, and evaluating emerging drugs targeting IL-33 and related pathways that influence both T2 and non T2 inflammation. At the same time, there is a need to revisit non-biologic strategies, such as macrolides, weight management, and treatment of metabolic and environmental “drivers” and to define their true disease modifying potential in children. The concept of treatable traits offers a pragmatic structure for implementing precision medicine by shifting the focus from diagnostic categories to specific, measurable targets such as airflow limitation, chronic infection, obesity, psychological burden, and environmental exposure. Evidence from severe asthma indicates that the systematic identification and management of these traits can improve disease control and reduce exacerbations [121,122]. Future efforts should adapt and validate these approaches for the pediatric population across the entire severity spectrum, not solely within tertiary care settings. Incorporating traits associated with T2 low asthma will be especially important for children who currently lack targeted biologic therapies. In the end, we must emphasize the need to rethink how studies are designed and which children are enrolled. Future studies must deliberately include younger age groups, under-represented endotypes (especially T2 low and mixed inflammation), and diverse ethnic and environmental backgrounds. This will help translate mechanistic insights into actionable, individualized care pathways that can be implemented into routine pediatric practice.

## 4. Conclusions

T2-low asthma in children emerges as a prevalent yet underrecognized endotype, distinguished by persistently low type 2 biomarkers, non-allergic clinical features, and strong associations with modifiable risk factors, including obesity, passive smoke exposure, and recurrent respiratory infections. This review underscores the heterogeneity within T2-low asthma, ranging from neutrophilic/Th17-high inflammation to paucigranulocytic airway remodeling and metabolically driven disease. It also highlights the critical diagnostic challenge posed by the suppression of T2 biomarkers induced by high-dose inhaled corticosteroid therapy or systemic steroids.

Pragmatic phenotyping algorithms using accessible tools such as blood eosinophil counts (<150–200/µL), fractional exhaled nitric oxide (FeNO; <20–25 ppb), and atopy testing offer a feasible first step in identifying probable T2-low asthma. This approach enables targeted management of comorbidities and helps avoid the use of ineffective T2-targeted biologics.

Since there is a significant gap in pediatric-specific longitudinal data, validated biomarkers for T2-low subendotypes, and randomized con-trolled trials evaluating emerging therapies, future efforts should prioritize systematic screening for T2-low phenotypes in routine pediatric asthma care, multicenter prospective cohort studies tracking endotype stability from preschool age through adolescence, and innovative clinical trials targeting non-T2 pathways to advance precision medicine for affected children. Future research must also integrate omics-based endo-typing, composite biomarker development, and treatable-traits-oriented care.

Upstream biologics targeting epithelial alarmins and non-T2 inflammatory pathways, together with refined use of existing therapies and non-pharmacological interventions, offer a realistic path toward genuinely personalized asthma management. Such an approach moves beyond a one-size-fits-all, eosinophil-centered paradigm and instead tailors treatment to each child’s inflammatory profile, comorbidities, and environmental exposures.

## Data Availability

No new data were created or analyzed in this study. Data sharing is not applicable to this article.

## Supplementary Materials

The following supporting information can be downloaded at: https://www.mdpi.com/article/10.3390/jcm15020907/s1, Figure S1: Low-T2 Asthma Pathophysiology; Figure S2: Diagnostic algorythm for differentiation between T2-high and T2-low asthma in childhood.

## Abbreviations

The following abbreviations are used in this manuscript:

T2	type 2
Ig	immunoglobulin
IL	interleukin
FeNO	fraction of exhaled nitric oxide
NHANES	National Health and Nutrition Examination Survey
NO	nitrogen dioxide
O_3_	ozone
PM_2–5_	fine particular matter
TNF-α	tumor necrosis factor-α
RSV	respiratory syncicial virus
NETs	neutrophil extracellular traps
TSLP	thymic stromal lymphopoietin
HMGB1	high-mobility group box 1 protein
MMP-9	matrix metalloproteinase-9
VEGF	vascular endothelial growth factor
fMLP	formyl-methionyl-leucyl-phenylalanine
ILCs	innate lymphoid cells
GCSF	granulocyte colony-stimulating factor
IRF 5	regulatory factor 5
ICS	inhaled corticosteroids
SABA	short-acting β_2_-agonists
GINA	Global Initivative for Asthma
NICE	National Institute for Health and Care Exellence
LAMAs	long-acting muscarinic antagonists
LABAs	long-acting β_2_-agonists
MABs	monoclonal antibodies

## Appendix A

**Table A1:** Prevalence of T2-low asthma in different studies.

Study	Population/Setting	How T2-Low or Non-T2 Defined	Reported Prevalence or Proportion of Th2-Low Asthma	Key Findings Relevant for Children with Th2-Low Asthma
NHANES 2007–2012 (USA, school-aged children 6–17 yrs) [20]	505 children with asthma	T2-low defined by AEC < 300 cells/µL and FeNO < 25 ppb (secondary thresholds also used)	45.7%	female, older, overweight/obese
ALLIANCE cohort (“T2-high asthma phenotypes across lifespan”, 2022) [21]	Mixed ages: preschool, school-age children, and adults with asthma (children total 473)	Phenotypes defined by blood eosinophils and allergen-specific IgE; “T2-low” = neither eosinophilia nor atopy; “T2-high” = eosinophilia + atopy; plus, other categories like eosinophilia-only or atopy-only.	0–2 yr—64.5% 3–5 yr—36.9% 6–8 yr—20.5% 9–11 yr—11.9% 12–14 yr—18.5% 15–17 yr—11.1%	With increasing age, T2-low tends to decrease; atopy or eosinophilia tends to become more common.
“Pediatric Asthma in Hospitalized Children—Exploring airway inflammation” (2024–2025) [22]	Hospitalized children with asthma, total 351 children included which could be classified as the known type of airway inflammation.	Classification using blood eosinophil counts, specific IgE (sIgE), and age stratification; thresholds like EOS 150 (Standard 1), 300 (Standard 2), 470 (Standard 3) cells/μL and sIgE ≥ 0.7 kU/L; defining groups: “Only-atopy”, “Only-EOS”, “T2-high”, “T2-low” (neither eosinophilia nor atopy)	Standard 1—19.4% Standard 2—25.6% Standard 3—28.2%	With increasing age, T2-low tends to decrease; atopy or eosinophilia tends to become more common. Findings indicated that patients with T2-low airway inflammation could have a longer time from symptoms onset to admission, a longer time for hospitalization, a lower proportion of atopic dermatitis, and a higher proportion of siblings.

**Table A2:** Potential T2-low asthma biomarkers in children.

Category	Biomarker	Comments/Phenotype	References
Non eosinophilic/neutrophilic marker	Sputum neutrophils	High neutrophil proportion associated with the T2-low phenotype	[1,2]
	IL-8 (CXCL8)	Elevated sputum levels in more severe disease	[3]
Systemic inflammatory markers	IL-6	Sporadically elevated in children; reflects low-grade systemic inflammation	[4,5]
	Leptin	Leptin modulates Th1/Th2 balance and Th17-driven non-T2 inflammation in obese children with asthma	[6]
Exhaled breath markers	FeNO	Low values (<25 ppb) suggest absence of T2 inflammation	[7]
Molecular/cytokine markers	IL-17	Th17 cytokine; associated with neutrophilic infiltration	[2]
Infectious/microbiome markers	Bacterial load in sputum	Elevated in T2-low exacerbations	[2]
	BAL neutrophils+ proteomics	Associated with infection and the T2-low phenotype	[8,9]
