# Supplementary material for: Beyond the Usual Suspects: Unmasking Low-T2 Asthma in Children
Source: J Clin Med. 2026 Jan 22;15(2):907. doi: 10.3390/jcm15020907 (PMC12842524; doi:10.3390/jcm15020907)
Supplement: Supplementary file 1 [file jcm-15-00907-s001.zip › jcm-4071949-supplementary.pdf]

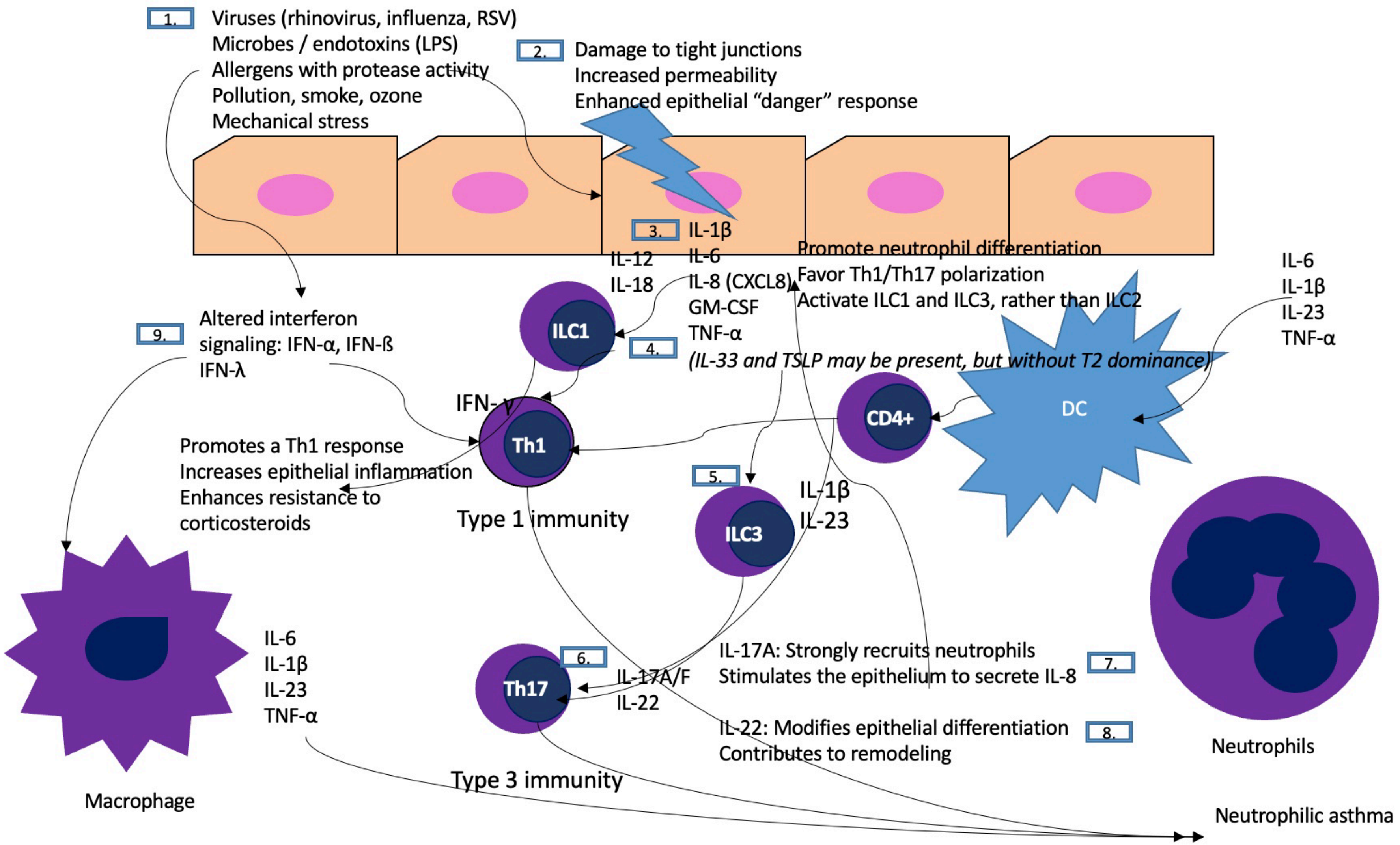

1.
Viruses (rhinovirus, influenza, RSV)
Microbes / endotoxins (LPS)
Allergens with protease activity
Pollution, smoke, ozone
Mechanical stress
2.
Damage to tight junctions
Increased permeability
Enhanced epithelial "danger" response
3.
IL-1β
IL-6
IL-8 (CXCL8)
GM-CSF
TNF-α
(IL-33 and TSLP may be present, but without T2 dominance)
IL-12
IL-18
Promote neutrophil differentiation
Favor Th1/Th17 polarization
Activate ILC1 and ILC3, rather than ILC2
IL-6
IL-1β
IL-23
TNF-α
ILC1
4.
9.
Altered interferon
signaling: IFN-α, IFN-ß
IFN-λ
IFN-γ
Th1
CD4+
DC
Promotes a Th1 response
Increases epithelial inflammation
Enhances resistance to
corticosteroids
Type 1 immunity
5.
ILC3
IL-1β
IL-23
IL-6
IL-1β
IL-23
TNF-α
Macrophage
6.
Th17
IL-17A/F
IL-22
IL-17A: Strongly recruits neutrophils
Stimulates the epithelium to secrete IL-8
7.
IL-22: Modifies epithelial differentiation
Contributes to remodeling
8.
Neutrophils
Type 3 immunity
Neutrophilic asthma

Figure S1: Low-T2 Asthma Pathophysiology

- Viruses (such as rhinovirus, influenza, and RSV), microbes (e.g., LPS), allergens with protease activity, pollution, smoke, ozone, and mechanical stress (1) contribute to the disruption of epithelial tight junctions (2), thereby increasing epithelial permeability and initiating a "danger" response. This response releases alarmins like IL-1β, IL-6, IL-8, and GM-CSF from epithelial cells (3). These alarmins activate ILC1 (4) and ILC3 cells (5), leading to the polarization of Th1 and Th17 cells. IL-6, IL-23, and TNF-α promote neutrophil differentiation, primarily through IL-17A/F (6), driving neutrophilic inflammation in the lungs (7). Additionally, IL-22 from Th17 cells contributes to airway remodeling (8).
- Impaired IFN signaling reduces the activation of Th1 cells and cytotoxic T cells, weakening the immune system's ability to control inflammation. This results in an over-recruitment of neutrophils, along with excessive activity of macrophages and neutrophils, causing chronic neutrophilic inflammation and resistance to corticosteroid treatment, a hallmark of low T2 asthma."

Abbreviations: RSV – Respiratory Syncytial Virus, LPS – Lipopolysaccharide, IL – Interleukin, IL-1β – Interleukin-1 beta, IL-6 – Interleukin-6, IL-8 (CXCL8) – Interleukin-8 / C-X-C motif chemokine ligand 8, IL-12 – Interleukin-12, IL-18 – Interleukin-18, IL-22 – Interleukin-22, IL-23 – Interleukin-23, IL-17A/F – Interleukin-17A and Interleukin-17F, GM-CSF – Granulocyte–Macrophage Colony-Stimulating Factor, TNF-α – Tumor Necrosis Factor alpha, IFN – Interferon, IFN-α – Interferon alpha, IFN-β – Interferon beta, Th1 – T helper type 1 cells, Th17 – T helper type 17 cells, ILC – Innate Lymphoid Cells, ILC1 – Type 1 Innate Lymphoid Cells, ILC2 – Type 2 Innate Lymphoid Cells, ILC3 – Type 3 Innate Lymphoid Cells, CXCL8 – C-X-C motif chemokine ligand 8, TSLP – Thymic Stromal Lymphopoietin, T2 – Type 2 immunity

Figure S2. Diagnostic algorythm for differentiation between T2-high and T2-low asthma in childhood

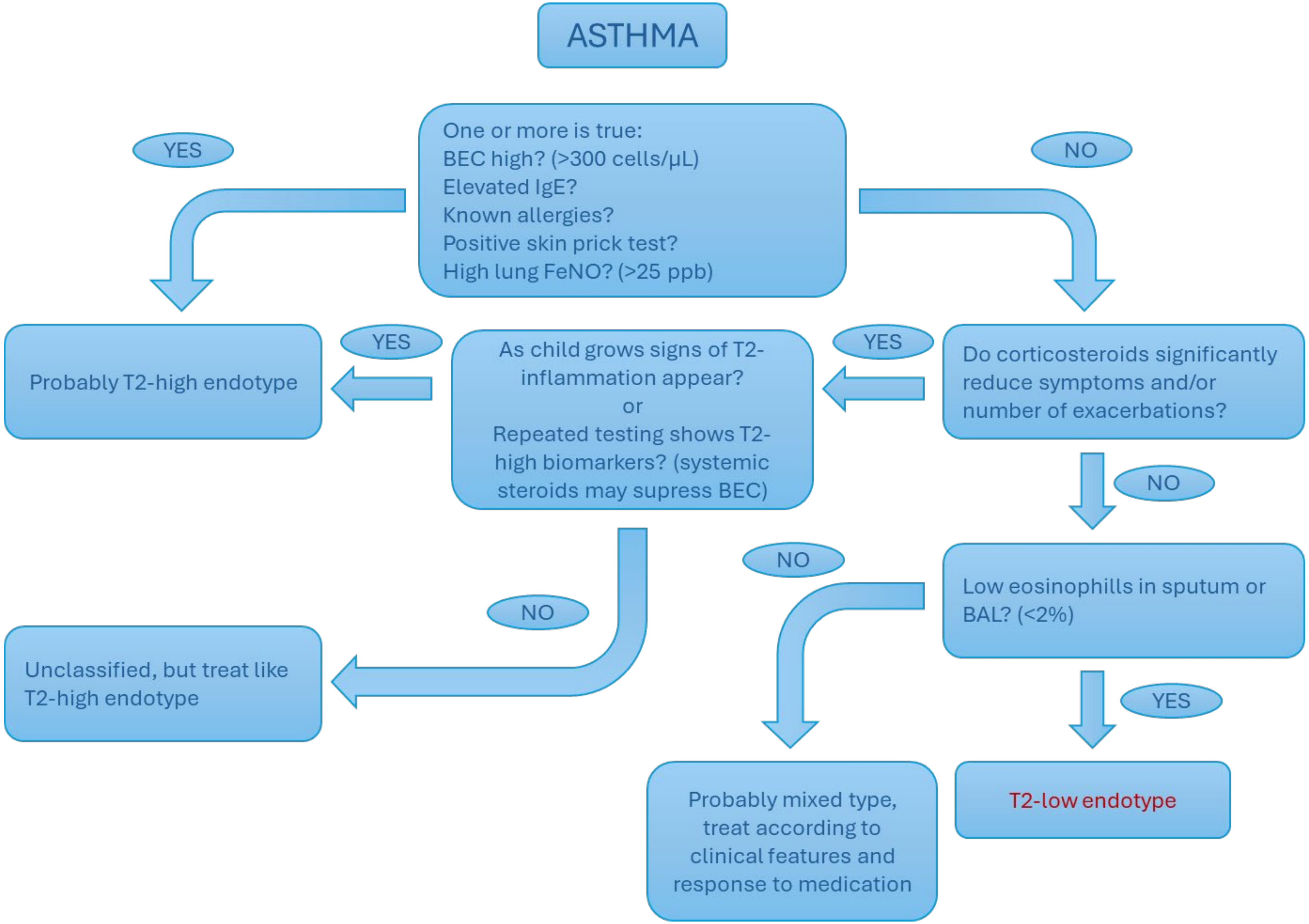

When assesing a child with suspected T2-low asthma the first step in the diagnostic approach should be whether there are signs or laboratory findings that would suggest otherwise. Therefore BEC, IgE levels, positive allergy tests and high lung FeNO would indicate an eosinophillic inflammation. In children the later onset of T2-high endotype markers can be misleading, but a good response ICS could differentiate between the need for further diagnostic evaluation along the T2-low endotype pathway.

In cases where systemic steroids were used, BEC may be supressed, while lung FeNO is mostly reduced with ICS so repeted testing after steroid withdrawal would be the preferred action before excluding T2-high endotype.

If there is no sign of eosinophillic inflammation or patient history, and inhaled corticosteroids are not useful in controling asthma symptoms, T2-low asthma is probable. To verify and subdivide T2-low asthma endotypes, sputum and BAL cytologic diagnosis should be performed.

Abbreviations: BEC – blood eosinophil count, ICS- inhaled corticosteroids, BAL – bronchoalveolar lavage
